# Between-bottle homogeneity test of new certified reference materials employing wavelength dispersive X-ray fluorescence spectrometry

**DOI:** 10.1186/s13065-019-0528-4

**Published:** 2019-02-12

**Authors:** Abubakr M. Idris

**Affiliations:** 1 Department of Chemistry, College of Science, King Khalid University, Abha, 61321 Saudi Arabia; 2 Research Center for Advanced Materials Science (RCAMS), King Khalid University, P.O. Box 9004, Abha, 61413 Saudi Arabia

**Keywords:** Certified reference material, Homogeneity test, XRF, Macro-elements, Micro-elements, Trace elements, Elemental analysis

## Abstract

**Background:**

A pilot study has being carried out at our laboratories, with international collaborators, to develop seven certified reference materials (CRMs), which have matrices of mainly soil and biological tissues. The CRMs will be certified for macro-, micro- and trace elements for environmental, toxicological, agronomic and nutritional purposes. Homogeneity of element concentrations is a critical step in the production process of CRMs. This work employs wavelength dispersive X-ray fluorescence spectrometry (WD-XRF) to test between-bottle homogeneity of the CRMs.

**Results:**

The relative standard deviation (RSD), relative average deviation, Skewness and Kurtosis of element contents in seven bottles out of 80 bottles of each CRM were considered to assess homogeneity in terms of variability and distribution. More than 50% of the number of quantified elements recorded RSD between 2 and 5%. Hence, based on an in-house classification, the intended CRMs recorded excellent to good between-bottle homogeneity. Nevertheless, the contents of some elements (Ni, Rb, Zn and Br) experienced high RSD values (> 10%). The Skewness and Kurtosis values of most elements are around one indicating symmetric distribution and thus have an absence of tailing relative to the normal distribution.

**Conclusions:**

WD-XRF provides fit for purpose data for assessment of initial between-bottle homogeneity in terms of rapidity, ease of use, multi-element quantification and sample non-destruction.
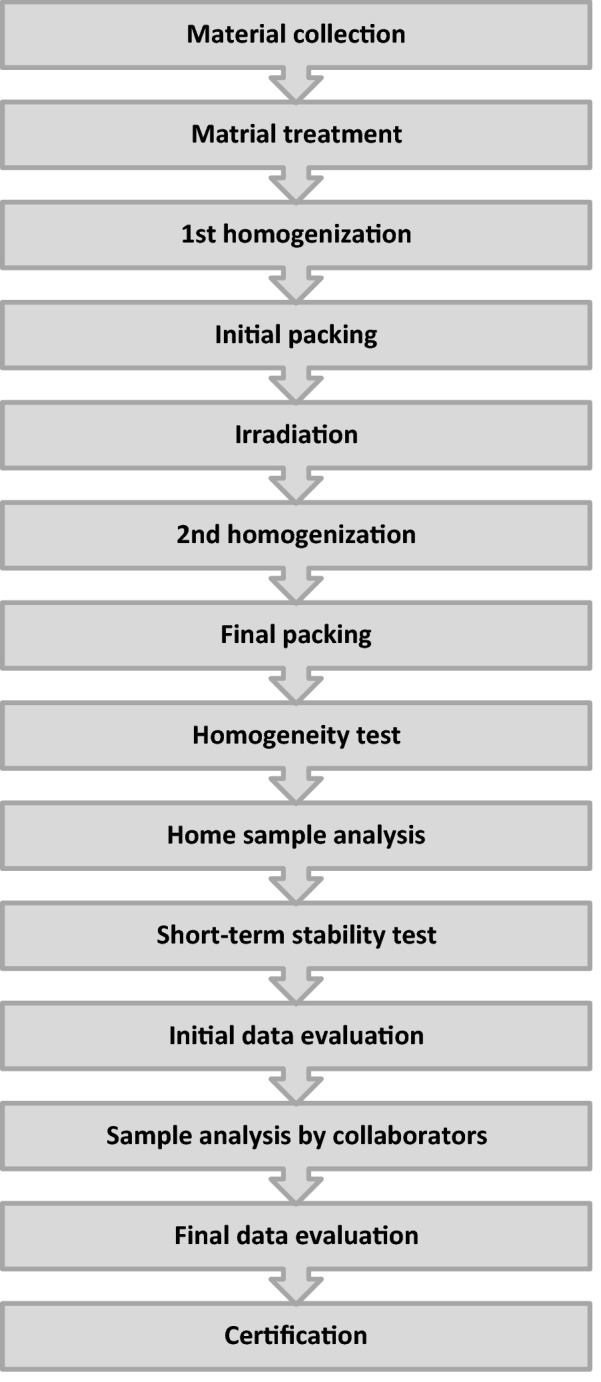

**Electronic supplementary material:**

The online version of this article (10.1186/s13065-019-0528-4) contains supplementary material, which is available to authorized users.

## Background

Material matrix is arguably the most critical factor controlling chemical analysis procedures including sample treatment and measurement. Reference materials (RMs) and certified reference materials (CRMs) play a vital role in the quality assurance of chemical analysis. As defined by the International Standards Organization (ISO) [1], an RM is “material, sufficiently homogeneous and stable with respect to one or more specified properties, which has been established to be fit for its intended use in a measurement process” while a CRM is “reference material characterized by a metrologically valid procedure for one or more specified properties, accompanied by a reference material certificate that provides the value of the specified property, its associated uncertainty, and a statement of metrological traceability”. Hence, CRMs could be recommended for laboratory accreditation, instrument calibration and suitability check of equipment, reagents and standards, in addition to training practitioners, checking infrequently used methods, troubleshooting, method validation and verification of the correct use of an analytical method.

The critical criterion of selecting an appropriate CRM for different applications is matrix composition and the levels of the certified properties. Numerous CRMs have been developed with various matrices and different levels of certified properties. The origins of CRMs are always from the environment of same region of the metrology institutes, who are always responsible for the production of CRMs. The European Commission Joint Research Centre, Health Consumers and Reference Materials (Belgium), National Institute of Standards and Technology (United States), National Research Council of Canada and National Institute of Metrology China have provided CRMs with a wide range of environmental and biological matrices and certified for numerous organic and inorganic properties. Nevertheless, there is no well documented CRMs of matrices from the Middle East, with the exception of some CRMs recently produced from National Metrology Institute of Turkey.

**Fig. 1:**
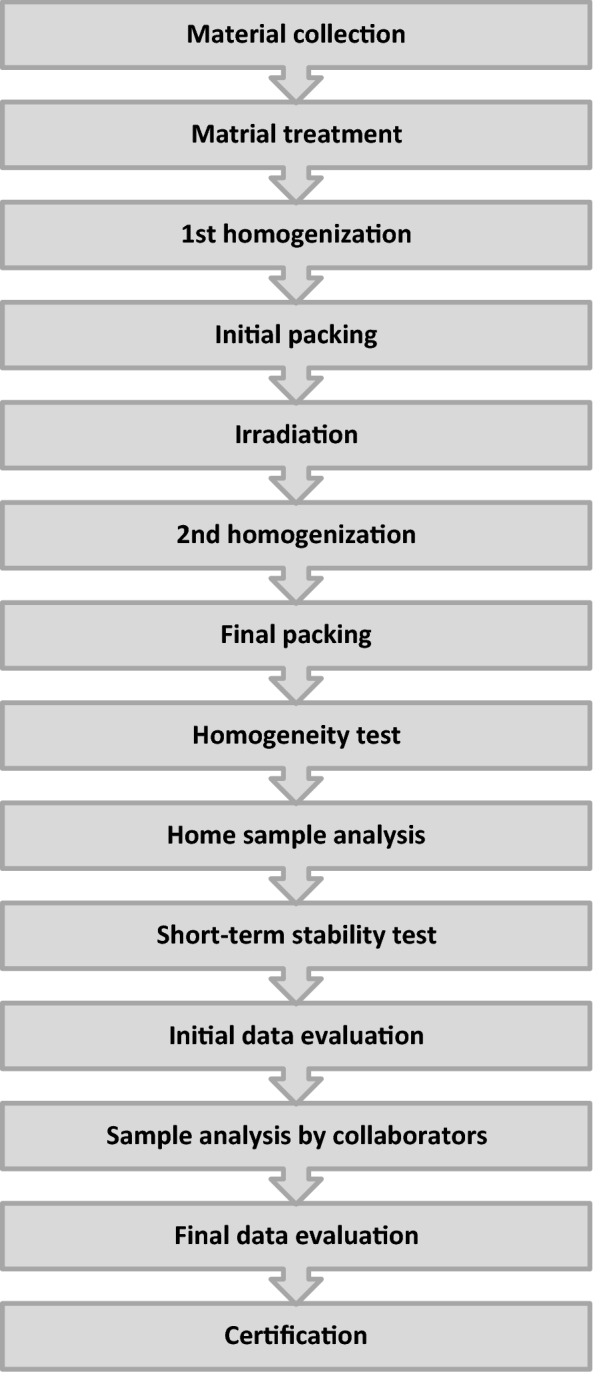
The process of the production of certified reference materials

Therefore, it has been proposed to carry out a pilot study on the production of CRMs of different matrices from the environment of Saudi Arabia. The study has been carried out at the laboratories of King Khalid University, Abha and sponsored by King Abdualziz City for Science and Technology, Riyadh. The study follows the processes as described in Fig. 1. The study has targeted the production of seven CRMs with the following codes and brief descriptions:

i. KACST401: surface soil from agricultural area in Alahssa farms, Eastern Region—to be certified for Al, Si, Cr, Mn, Fe, Co, Ni, Cu, Zn, As, Cd and Pb.
ii. KACST402: surface soil from the Third Industrial Area, Jeddah city—to be certified for Al, Si, Cr, Mn, Fe, Co, Ni, Cu, Zn, As, Cd and Pb.
iii. KACST404: urban street dust from Riyadh city—to be certified for Al, Si, Cr, Mn, Fe, Co, Ni, Cu, Zn, As, Cd and Pb.
iv. KACST403: surface coastal sediment from Aljubail harbor, Arabian Gulf—to be certified for Al, Si, Cr, Mn, Fe, Co, Ni, Cu, Zn, As, Cd and Pb.
v. KACST301: dates fruit (*Phoenix dactylifera* L.) from Alahssa farms, Eastern Region—to be certified for Na, Mg, P, K, Ca, Cr, Mn, Fe, Cu, Zn, Cd and Pb.
vi. KACST302: leaves of date palm (Phoenix dactylifera L.) from Alahssa farms, Eastern Region—to be certified for Na, Mg, P, K, Ca, Cr, Mn, Fe, Cu, Zn, Cd and Pb.
vii. KACST201: edible fish muscle of Greasy Grouper (*Epinephelus tauvina* sp.) from Aljubail fisheries, Arabian Gulf—to be certified for Cr, Mn, Fe, Co, Ni, Cu, Zn, As, Cd and Pb.

Among the steps of the production process of CRMs, homogeneity is a critical step. Homogeneity is required to establish that the degree of homogeneity is fit for purpose. The homogeneity should be reported in the certificate of a CRM as material uncertainty. Hence, testing homogeneity is essential for the production of CRMs [2]. It is recommended to test homogeneity in term of the certified properties. As the proposed CRMs in the current study will be certified for some macro-, micro- and trace elements, inductively coupled plasma-mass spectrometry (ICP-MS) and instrumentation neutron activation analysis (INAA) are recommended as the most reliable and precise techniques for elemental analysis [3, 4]. However, ICP-MS requires critical sample treatment procedures while INAA is expensive technique and requires critical precautions for safety. A fast, simple, reliable and precise analytical technique for element analysis in various matrices and at different levels is desirable for testing the homogeneity even at initial step.

Despite X-ray fluorescence (XRF) is arguably a semi-quantitative technique, it was exploited for the certification of CRMs of various matrices and different levels of certified properties. Basically, the instrumentation of XRF consists of an excitation source, optical components for shaping and guiding X-ray beam to the sample and a detection device for analyzing and constructing spectrum [5]. Based on different types of their major components, various XRF techniques have been introduced. Generally, wavelength-dispersive (WD-XRF), which is the oldest approach, and energy-dispersive XRF are the main groups. While ED-XRF utilizes detectors that are able to discriminate the energy of the X-rays reaching the detector, WD-XRF utilizes a crystal analyzer resulting in high-energy resolution and sensitivity. Namely, WD-XRF provides higher precision, which is the targeted feature is this study, besides high accuracy and resolution [5, 6].

The most striking feature of XRF analysis is that the technique permits nondestructive analysis and hence there is no need for sample digestion and just simple treatment process is required for samples; a feature that minimizes analytical errors. XRF technique also allows qualitative and quantitative analysis at high levels of accuracy and precisions for almost all the elements; from Be to U and at different levels and in different matrices [7–9]. Additionally, XRF technique features simultaneous multi-element capacity and the analysis requires only a short irradiation time resulting in high sample throughput at low running costs. Furthermore, the technique records a wide dynamic range of concentrations covering up to nine orders of magnitude as well as low detection limits. Hence, the technique is appropriate for applications in many fields of science, research and quality control [10]. In contrast, the technique has the limitation of the absorption of low energy X-ray, which is emitted by low-Z elements, inside the sample itself [7–9]. Accordingly, XRF could be a satisfactory approach for testing the homogeneity of CRMs, in addition to its contribution in the certification of macro- and micro-elements. Satisfactory results were reported for the use of XRF for homogeneity tests of CRMs of different matrices including sediment [11], chromium ores [12], nickel ores [13], maize grain [14], ash of municipal solid waste [15] and crude oil [16]. Based on the above discussion, the aim of this study was to use WD-XRF for testing initial between-bottle homogeneity of seven CRMs benefitting the above-mentioned advantages of the technique.

## Experimental

### Preparation of certified reference materials

Different amounts of materials were collected based on the targeted quantity of one batch to be produced for the purpose of a pilot study. The following quantities of materials were collected 20 kg of KACST401, KACST402, KACST404 and KACST403; 50 kg of KACST301 and KACST302; 100 kg of KACST201. For the CRMs of soil matrices (KACST401, KACST402, KACST404 and KACST403), extraneous particles such as stones, roots, waste, etc. were manually eliminated. The materials were dried and sieved to obtain particle-size ≤ 1000 µm [17–19] with the exception of KACST404 (dust) that was sieved to obtain 100 µm [20, 21]. Materials of dates fruit matrix (KACST301) and date palm leave matrix (KACST302) were washed by distilled deionized water to remove dust. Kernels were removed from dates fruit. Dates fruit and leaves were cut into convenient parts to facilitate handling. Edible muscles of fish (KACST201) were separated and cut into smaller parts. Dates fruit, leaves and fish were freeze dried and ground. For dates fruit, leaves and fish, particle-size of ≤ 850, ≤ 500 and ≤ 200 µm were separated by dry sieving, respectively.

Two homogenization steps, i.e. before and after the irradiation step, were carried out in a 50 L mixer for 12 h for each step. The container and the paddles of the mixer were made of stainless steel and coated with polyethylene sheets. The irradiation process was carried out using Co^60^ facility, Nordion Gamma Cell 220 (Nordion, Canada Ltd.). The dose rate for each sample was 4.1 kGy/h at 25 kGy of absorbed dose (1 Gy = 100 rad = 1 J kg^−1^). The irradiation treatment was performed in vessels of Pyrex-glass at room temperature. The irritation dose is suitable for the preservation of CRMs.

**Fig. 2:**
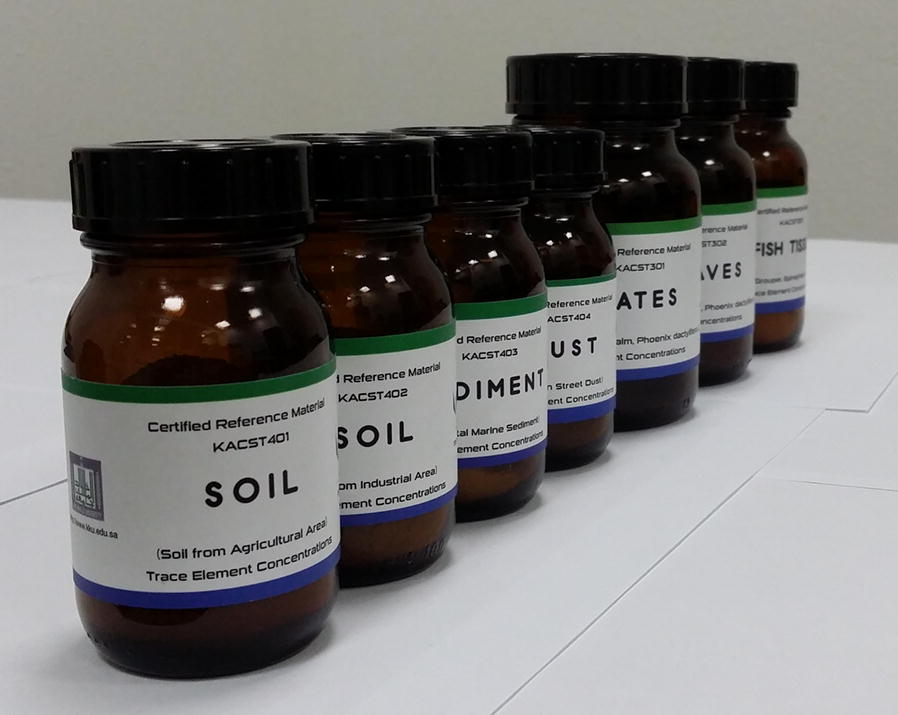
Certified reference materials

One batch of each CRM will be produced for the purpose of a pilot study. The sample size of each CRM of soil matrices was about 4 kg while that of biological matrices was about 2.4 kg. CRMs were packed in 150 mL and 250 mL brown bottles (Fig. 2), resulting in 80 bottles of each CRM. Each bottle of a CRM of soil matrix contained 50 g while that of CRM of biological matrices contained 30 g. Between-bottle homogeneity test was carried out for seven bottles of each CRM. Random stratified sample picking scheme was applied for bottle selection.

### Measurements by WD-XRF

Each sample collected from a bottle was measured in triplicate. The samples were ground to have particle-size of < 100 µm, mixed with boric acid and pressed in a briquetting die at 30 tons to form a standard 40 mm puck. The tests were run in a Panalytical PW 2403 Magix Wavelength Dispersive X-ray Fluorescence Spectrometer using Rh radiation under vacuum. The instrument is a sequential spectrometer that optimizes the test conditions for each element, ranging from Na and U, to enhance the sensitivity and precision.

The WD-XRF spectrum were evaluated using the Fundamental Parameters standardless quantification software associated with the XRF system. This approach uses established sensitivity factors for pure elements and takes into account fluorescence yield, absorption and enhanced excitation effects. Three CRMs were used to test the recovery of measurements. The CRMs were developed by NIST, which included Montana II Soil (2711a), Inorganics in Marine Sediment (2702) and Slurried Spinach (2385).

## Results and discussion

Primarily, the triplicate measurements of each sample recorded variation with relative standard deviation (RSD) values of < 1.0% of almost all examined elements in all matrices of CRMs, indicating good repeatability of WD-XRF measurements. This result matches the results obtained from measurements of CRMs of soil matrices by WD-XRF [22]. However, Rouillon and Taylor [23] reported repeatability in the range of 0.2–10% for measurements of different elements obtained by portable-XRF. Rydberg [24] also reported RSD values of < 5% for a wide range of elements with the exception of Cu (6%), As (8%), Br (18%), Zr (6%), Ba (9%), W (10%), Sc (10%), Cr (21%), Co (6%), Br (15%) and Sn (13%). The recovery of CRMs from NIST recorded a range of 94–115%, indicating acceptable accuracy.

For assessment of between-bottle homogeneity of the current CRMs, we suggested four homogeneity levels based on the RSD values of contents of 50% of the number of quantified elements as follows: RSD < 2%—excellent homogeneity; RSD 2–5%—good homogeneity; RSD 5–10%—acceptable homogeneity; RSD > 10%—rejected homogeneity. However, other variability indices including relative average deviation (RAD), Skewness and Kurtosis were also considered in this study. Despite both RSD% and RAD% measure variability, the RAD uses absolute values instead of squares to circumvent the issue of negative differences between data and the average. Skewness indicates for the lack of symmetry while Kurtosis indicates for whether the data are heavy-tailed or light-tailed relative to the normal distribution [25].

Pearson correlation coefficients between the average of element contents, atomic number (Z) of element, RSD and RAD were calculated for three CRMs of different matrices, i.e. soil, leaves and fish muscle. The matrices are compiled in Table 1. No significant correlation was observed between element content, RSD and RAD, suggesting good stability of WD-XRF for the measurements of elements at low contents. Furthermore, no significant correlation was observed between Z, RSD and RAD, suggesting also good stability of WD-XRF for the measurements of elements with low-Z.

**Table 1:** Pearson correlation matrices between atomic number (Z), average of elements contents (C), relative standard deviation (RSD) and relative average deviation in three matrices of CRMs (soil, plant and animal tissue)

	Z	C	RSD	RAD
KACST401				
Z	1.000			
C	− 0.305	1.000		
RSD	0.138	− 0.268	1.000	
RAD	0.137	− 0.282	0.995	1.000
KACST302				
Z	1.000			
C	− 0.319	1.000		
RSD	0.426	− 0.303	1.000	
RAD	0.424	− 0.301	0.998	1.000
KACST201				
Z	1.000			
C	− 0.224	1.000		
RSD	0.349	− 0.253	1.000	
RAD	0.342	− 0.251	1.000	1.000

The descriptive statistics of element contents (weight%) in KACST401 is presented in Table 2. Twenty elements were successfully quantified and a wide range of contents (0.002–24.086%) was observed. WD-XRF demonstrated accurate and precise element contents at different levels in soil matrices [22, 26]. The macro-elements (> 1% contents) in KACST401 were in the following descending order: Si ≫ Ca > Al > S > K while the micro-elements (1–0.1% contents) were in the following descending order: Fe > Na > Cl > Ti. High content of Si reflects that the majority of the matrix of KACST401 is silicates. Ten elements recorded RSD values of < 2.0%, indicating excellent between-bottle homogeneity based on the classification mentioned above. Ni recorded the highest RSD value (11.35%) and the highest RAD (8.44%). Hence, Ni measurements demands treatment. The five lowest RSD values were recorded for Al, Ba, Ca, Cl and Na while the five lowest RAD values were recorded for Al, Ba, Cl, Na and Si. In general, the order of variability in between-bottle element contents based on RSD and RAD was almost similar. On the other hand, the Skewness values of other elements were approximately < 1 indicating symmetrical distribution of element contents, i.e. the distribution looks the same to the left and the right of the average. However, the Skewness of Ni was 0.21, indicating symmetrical distribution while the Kurtosis was − 0.47, indicating low level of tailing toward low contents. In general, most Kurtosis values were around one and two and with negative mode indicating, light- to heavy-tailed relative to the normal distribution.

**Table 2:** Descriptive statistics of elements contents (weight%) in KACST401 CRM

Element	Average	SD	RSD%	RAD%	Skewness	Kurtosis
Al	2.609	0.0107	0.41	0.28	1.52	2.71
Ba	0.030	0.0002	0.74	0.60	0.63	− 1.04
Br	0.002	0.0001	3.29	2.33	–	–
Ca	8.763	0.0640	0.73	0.66	0.42	− 2.45
Cl	0.228	0.0010	0.42	0.36	0.76	− 1.69
Cr	0.020	0.0014	7.05	6.11	0.31	− 1.93
Fe	0.855	0.0228	2.66	2.24	− 0.10	− 1.51
K	1.130	0.0173	1.53	1.26	0.00	− 1.98
Mg	1.073	0.0138	1.29	1.10	0.36	− 2.09
Mn	0.014	0.0009	6.61	5.19	0.13	− 0.26
Na	0.313	0.0018	0.57	0.50	0.30	− 2.15
Ni	0.013	0.0015	11.4	8.44	0.21	− 0.47
P	0.039	0.0018	4.78	3.93	− 0.51	− 1.48
Rb	0.004	0.0002	3.96	3.48	− 0.38	− 2.11
S	1.511	0.0168	1.11	0.86	− 0.31	− 1.47
Si	24.08	0.2116	0.88	0.64	1.44	2.08
Sr	0.032	0.0006	1.77	1.57	0.51	− 2.15
Ti	0.122	0.0027	2.24	1.97	− 0.21	− 2.38
Zn	0.004	0.0001	4.19	3.31	0.26	− 0.97
Zr	0.023	0.0004	1.57	1.21	0.98	0.69

*SD* standard deviation, *RSD* relative standard deviation, *RAD* relative average deviation

The macro-elements in KACST403 (Table 3) were in the following descending order: Si ≫ Fe > Al > Ca > Mg > Na. High levels of Si and Fe suggest the combination matrix of silicate and hematite. As KACST403 is soil from industrial area, high levels of Fe may also be due to anthropogenic contribution from industrial activity. Twenty-one elements were quantified in KACST403. Fourteen elements recorded RSD values of < 2%, indicating excellent homogeneity, as in KACST401. The same 14 elements also recorded the lowest RAD values. The Skewness values of all elements were < 1.00 with the exception of K, Pb and Sr, indicating symmetrical distribution. Notably, the same exceptional elements recorded positive Kurtosis values of 6.41, 4.69 and 3.68, respectively, indicating very heavy-tailed toward high contents (Additional file 1).

**Table 3:** Descriptive statistics of elements contents (weight%) in KACST402 CRM

Element	Average	SD	RSD%	RAD%	Skewness	Kurtosis
Al	6.153	0.0450	0.73	0.61	− 0.20	− 1.86
Ba	0.051	0.0019	3.70	2.78	0.82	0.28
Ca	4.047	0.0382	0.94	0.77	0.84	− 1.29
Cl	0.592	0.0085	1.44	1.20	− 0.26	− 1.57
Cr	0.018	0.0011	6.22	5.55	0.36	− 2.32
Cu	0.009	0.0007	8.09	6.39	0.40	− 0.80
Fe	5.406	0.0223	0.41	0.33	0.63	− 1.04
K	1.424	0.0960	6.74	4.31	2.50	6.41
Mg	1.860	0.0100	0.54	0.46	0.00	− 2.60
Mn	0.088	0.0016	1.84	1.46	0.77	− 1.26
Na	1.160	0.0082	0.70	0.49	0.00	− 1.20
Ni	0.013	0.0001	0.73	0.58	− 0.28	0.04
P	0.079	0.0144	18.3	12.2	− 2.11	4.69
Pb	0.012	0.0009	7.47	4.78	2.22	5.27
Rb	0.004	0.0002	4.78	3.90	− 0.29	− 1.45
S	0.328	0.0050	1.53	1.21	0.70	− 0.06
Si	19.61	0.1574	0.80	0.67	0.04	− 1.68
Sr	0.041	0.0002	0.57	0.40	1.84	3.68
Ti	0.473	0.0088	1.86	1.42	0.93	− 0.11
Zn	0.344	0.0031	0.90	0.81	0.28	− 2.47
Zr	0.027	0.0001	0.50	0.38	− 0.35	− 0.30

*SD* standard deviation, *RSD* relative standard deviation, *RAD* relative average deviation

As shown in Table 4, Ca was found the dominant element (25%) in KACST404, suggesting calcareous matrix of dust from Riyadh city, Saudi Arabia. As in KACST401, Ni recorded the highest RSD value (11.56%). Twenty elements were quantified in KACST404. XRF has proven to be efficient tool for the detection of a wide range of heavy metals in dust samples [27–29]. In the current study, all elements recorded RSD values of < 2%, with the exception of Cu, Rb and Zn that recorded RSD values of 4.788.10 and 4.29, respectively. This result indicates excellent between-bottle homogeneity, as of KACST401 and KACST403. The Skewness values showed symmetrical distribution since all positive and negative values were < 1.0, with the exception of K and Ni that recorded Skewness values of 1.11 and 1.23, respectively. Notably, negative tailing was recorded for Cu (2.32), Fe (2.49) and P (2.11).

**Table 4:** Descriptive statistics of elements contents (weight%) in KACST404 CRM

Element	Average	SD	RSD%	RAD%	Skewness	Kurtosis
Al	1.780	0.0082	0.46	0.32	0.00	− 1.20
Ca	24.76	0.1134	0.46	0.38	0.24	− 1.23
Cl	0.368	0.0020	0.55	0.48	0.13	− 1.96
Cr	0.034	0.0006	1.86	1.52	0.08	− 1.77
Cu	0.021	0.0010	4.78	4.27	− 0.45	− 2.32
Fe	2.467	0.0325	1.32	1.17	− 0.25	− 2.49
K	0.604	0.0008	0.13	0.11	1.11	0.27
Mg	0.865	0.0082	0.94	0.82	− 0.58	− 1.77
Mn	0.036	0.0002	0.60	0.48	− 1.11	0.86
Na	0.358	0.0012	0.34	0.27	− 1.15	− 0.06
Ni	0.027	0.0031	11.6	10.1	− 0.23	− 2.24
P	0.079	0.0002	0.22	0.19	0.38	− 2.11
Pb	0.015	0.0001	0.59	0.48	0.35	− 1.82
Rb	0.004	0.0003	8.10	6.29	− 0.26	− 0.89
S	0.612	0.0068	1.10	0.89	− 0.44	− 1.50
Si	8.950	0.0764	0.85	0.67	0.86	− 0.87
Sr	0.042	0.0003	0.65	0.55	0.00	− 2.31
Ti	0.331	0.0012	0.35	0.26	0.91	− 0.15
Zn	0.037	0.0016	4.29	3.41	− 0.51	− 0.90
Zr	0.082	0.0008	0.92	0.76	0.25	− 1.42

*SD* standard deviation, *RSD* relative standard deviation, *RAD* relative average deviation

As in KACST404, high level of Ca (22.5%) was recorded in KACST403 (Table 5). This result suggests that the matrix of sediment from the Arabian Gulf is calcareous as agrees with a previous result published elsewhere [30]. Ten elements out of 18 recorded RSD values of < 2.0 indicating excellent between-bottle homogeneity as in the above-mentioned CRMs. Symmetric distribution was also observed since all Skewness values were < 1, with the exception of Fe (2.54). It was reported that the RSD values of repeatability of trace elements measurements in calcareous rocks by WD-XRF increased as concentrations increased [31]. In that study [31], the RSD values ranged from 2.5 to 55% for concentrations ranged from 1 to 100 µg g^−1^.

**Table 5:** Descriptive statistics of elements contents (weight%) in KACST403 CRM

Element	Average	SD	RSD%	RAD%	Skewness	Kurtosis
Al	1.756	0.0098	0.56	0.44	− 0.28	0.04
Br	0.004	0.0004	9.90	6.71	− 1.68	3.37
Ca	22.57	0.0488	0.22	0.18	− 1.23	− 0.84
Cl	1.042	0.0185	1.77	1.26	0.15	0.03
Cr	0.048	0.0039	8.19	6.38	− 0.68	− 0.07
Fe	1.026	0.0335	3.26	2.09	2.54	6.55
K	0.818	0.0021	0.25	0.22	− 0.17	− 2.10
Mg	1.199	0.0069	0.58	0.41	0.17	0.34
Mn	0.030	0.0010	3.24	2.85	− 0.37	− 2.13
Na	1.121	0.0121	1.08	0.91	0.41	− 1.53
Ni	0.014	0.0012	8.56	6.77	0.42	− 1.36
P	0.026	0.0012	4.79	3.62	− 0.45	− 1.22
Rb	0.004	0.0002	6.12	4.93	− 0.12	− 0.77
S	0.198	0.0034	1.72	1.48	− 0.20	− 2.20
Si	11.57	0.0488	0.42	0.35	− 1.23	− 0.84
Sr	0.308	0.0011	0.36	0.29	− 0.25	− 0.94
Ti	0.190	0.0008	0.43	0.30	0.00	− 1.20
Zn	0.003	0.0007	20.7	16.9	0.16	− 1.76

*SD* standard deviation, *RSD* relative standard deviation, *RAD* relative average deviation

Unlike CRMs of soil matrices, only 13 elements were quantified in KACST301 (Table 6), which could be attributed to low levels of various elements in dates fruit. Eight elements recorded RSD values < 5%, indicating good between-bottle homogeneity test. However, Ca, Cl, Cu K, Mg, P and S recorded RSD values of about 2% or less. It was reported that WD-XRF produces constant intensity when particle-size of plant tissue is less than 710 µm and pressed into pellets obtaining particle-size less than 500 µm [5, 32]. Furthermore, boric acid as a binder featuring-high purity, low X-ray absorption and good stability is useful for constant intensity [33]. Both small particle-size and the use of a selective binder improve repeatability, which is the targeted feature in this study. However, KACST301 also recorded less homogeneity level than CRMs of soil matrices. High levels of carbohydrates in dates fruit cause conglomerate of particles. All kurtosis values recorded ≈ 1 with negative mode, indicating negative tailing.

**Table 6:** Descriptive statistics of elements contents (weight%) in KACST301 CRM

Element	Average	SD	RSD%	RAD%	Skewness	Kurtosis
Al	0.0048	0.0009	19.1	16.0	− 1.23	− 0.82
Ca	0.5953	0.0005	0.08	0.07	1.23	− 0.84
Cl	0.4019	0.0097	2.42	2.03	− 0.46	− 1.68
Cu	0.0141	0.0004	2.59	2.06	0.12	− 1.16
Fe	0.0489	0.0112	23.0	18.2	− 0.96	− 0.57
K	2.5771	0.0550	2.13	1.76	0.66	− 1.30
Mg	0.0567	0.0008	1.36	1.12	− 0.25	− 1.28
P	0.0815	0.0017	2.09	1.76	0.07	− 1.91
Rb	0.0080	0.0003	3.76	3.13	− 0.83	− 1.28
S	0.0809	0.0002	0.21	0.17	− 0.62	− 1.40
Si	0.0454	0.0145	31.9	20.6	2.58	6.75
Sr	0.0091	0.0011	11.6	9.27	0.21	− 1.03
Zn	0.0068	0.0013	18.7	14.7	0.33	− 0.74

*SD* standard deviation, *RSD* relative standard deviation, *RAD* relative average deviation

As shown in Table 7, 17 elements were quantified in KACST302, unlike KACST301. This result could be attributed to higher element contents in KACST302 than KACST301 despite they were from the same plant species, i.e. date palm tree (*Phoenix dactylifera* L.). Macro- (P, K, Ca, Mg and S) and micro-nutrients (Fe, Cu, Mn, Zn, Cl and Ni), which are required for plant growth and increasing crop yields, were all detected in KACST302 [5]. Beneficial elements (e.g., Al, Na and Si) [5], which promote growth, were detected as well. The flowing descending order of element contents were recorded in KACST302, which ranged from 0.10% to 6.4: Si > Ca > Cl > K > S > Fe > Mg > Al > Na. Other elements (Br, Cu, Mn, Ni, P, Sr, Ti and Zn) recorded contents of < 0.1%. WD-XRF recorded reparability with RSD values of < 2 for determination of Ca and P in mineral supplements for cattle [7]. In the current study, 11 elements out of 17 in KACST302 recorded RSD values < 2%, indicating excellent between-bottle homogeneity. Notably, three elements (Cu, Sr and Ti) recorded RSD values between 2 and 5%. Hence, the homogeneity of KACST302 was better than KACST301, which may be due the absence of carbohydrate in the frontal.

**Table 7:** Descriptive statistics of elements contents (weight%) in KACST302 CRM

Element	Average	SD	RSD%	RAD%	Skewness	Kurtosis
Al	0.146	0.0020	1.34	1.09	− 0.29	− 1.45
Br	0.021	0.0001	0.25	0.23	− 0.37	− 2.80
Ca	3.350	0.0082	0.24	0.17	0.00	− 1.20
Cl	1.147	0.0049	0.43	0.36	− 1.23	− 0.84
Cu	0.018	0.0008	4.67	3.94	− 0.14	− 1.78
Fe	0.291	0.0025	0.86	0.67	0.90	− 0.17
K	0.976	0.0042	0.43	0.38	− 0.40	− 2.31
Mg	0.240	0.0012	0.49	0.37	0.67	− 0.45
Mn	0.033	0.0048	14.6	13.3	− 0.36	− 2.77
Na	0.102	0.0005	0.48	0.40	− 1.23	− 0.84
Ni	0.017	0.0028	16.76	13.6	− 1.11	− 0.59
P	0.095	0.0014	1.51	1.28	− 0.13	− 1.97
S	0.542	0.0033	0.61	0.51	− 0.14	− 1.91
Si	6.404	0.0079	0.12	0.10	− 1.11	0.27
Sr	0.016	0.0006	4.04	3.51	− 0.55	− 1.85
Ti	0.027	0.0008	2.98	2.24	0.17	− 0.99
Zn	0.014	0.0012	8.28	7.05	− 0.30	− 1.64

*SD* standard deviation, *RSD* relative standard deviation, *RAD* relative average deviation

Fourteen elements were quantified in KACST201 (Table 8). Seven elements recorded RSD values of < 2%. Hence, the between-bottle homogeneity of KACST201 is considered excellent to good. On the other hand, the following descending order of element contents (6–0.1%) was found: K > S > P > Cl > Ca > Na > Mg. Among other CRMs, As was quantified only in KACST201. The detected level of As (0.0099%) in fish muscle could be attributed to anthropogenic contribution in the Arabian Gulf, which has witnessed heavy oil industry and shipping activates [33].

**Table 8:** Descriptive statistics of elements contents (weight%) in KACST201 CRM

Element	Average	SD	RSD%	RAD%	Skewness	Kurtosis
Al	0.0047	0.0007	15.57	13.37	0.25	− 2.00
As	0.0099	0.0045	45.62	37.74	0.92	− 0.56
Br	0.0159	0.0003	1.78	1.39	− 0.57	− 0.55
Ca	0.6066	0.0300	4.95	3.75	0.17	− 0.43
Cl	1.2443	0.0079	0.63	0.49	1.76	2.36
Fe	0.0233	0.0010	4.26	3.41	− 0.39	− 1.17
K	6.1057	0.0237	0.39	0.31	0.30	− 1.46
Mg	0.1866	0.0010	0.52	0.42	− 0.28	0.04
Na	0.3049	0.0034	1.11	0.98	0.17	− 2.41
P	1.3471	0.0111	0.83	0.67	− 0.25	− 0.94
S	1.9457	0.0223	1.14	0.99	− 0.13	− 2.22
Si	0.0355	0.0012	3.33	2.67	0.20	− 1.11
Sr	0.0067	0.0004	5.61	4.60	− 0.76	− 1.29
Zn	0.0161	0.0005	3.06	2.34	− 0.23	− 0.57

*SD* standard deviation, *RSD* relative standard deviation, *RAD* relative average deviation

**Fig. 3:**
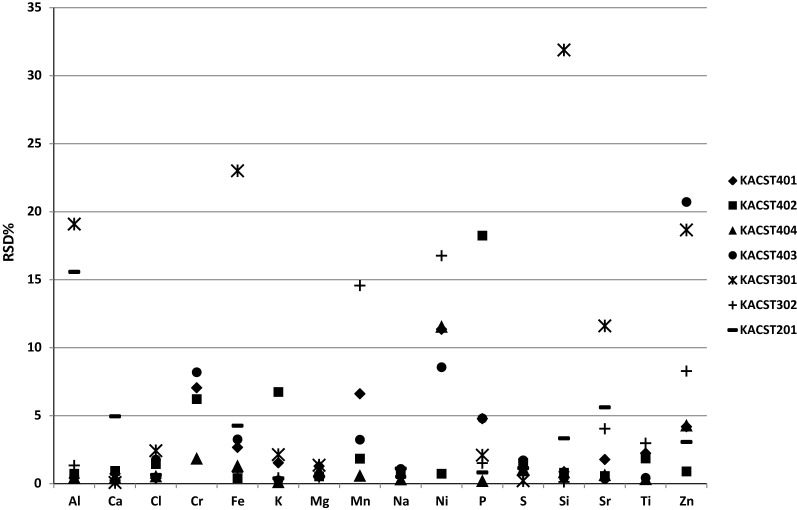
Relative standard deviation (RSD%) of homogeneity test

The RSD values of elements quantified in almost all CRMs are depicted in Fig. 3. The plot shows about 112 values. In general, the dominant RSD values were < 5%. Eight RSD values were in the range of 5–10% and other eight RSD values were in the range of 10–20% while only three RSD values were in the range of 20–35%. Most of the light elements (Ca, Mg, Na and S) recorded RSD values in all CRMs of ≤ 5%. This results suggest constant measurement of light elements by WD-XRF. Fe and Si also recorded RSD values of < 5% in all CRMs, with the exception of KACST301 (dates fruit). Eventually, the WD-XRF measurements demonstrated initially excellent between-bottle homogeneity of all CRMs, with the exception of KACST301 (dates fruit) that demonstrated excellent to good between-bottle homogeneity.

## Conclusion

WD-XRF offers useful analytical data for testing homogeneity of CRMs in terms of measurements of macro- and micro-elements including both certified and indicative properties. WD-XRF provides the advantages of multi-element and non-destructive analysis, besides simplicity and rapidity. Hence, this study fosters the use of non-destructive techniques, such as X-ray diffraction spectrometry, particle induced X-ray emission, laser-induced breakdown spectroscopy, for testing homogeneity in terms of either certified properties or indicative properties. Notwithstanding, more sensitive and reliable elemental analysis techniques are requested for confirmatory homogeneity test in term of certified properties.

## Additional file

**Additional file 1: KACST401.** Raw data and statistical analysis of KACST401 CRM. **KACST402**. Raw data and statistical analysis of KACST402 CRM. **KACST404**. Raw data and statistical analysis of KACST404 CRM. **KACST403**. Raw data and statistical analysis of KACST403 CRM. **KACST301**. Raw data and statistical analysis of KACST301 CRM. **KACST302**. Raw data and statistical analysis of KACST302 CRM. **KACST201**. Raw data and statistical analysis of KACST201 CRM.
